# Prognostic significance of diastolic blood pressure in patients with heart failure with preserved ejection fraction

**DOI:** 10.1007/s00380-021-01788-0

**Published:** 2021-02-02

**Authors:** Aya Fuchida, Sho Suzuki, Hirohiko Motoki, Yusuke Kanzaki, Takuya Maruyama, Naoto Hashizume, Ayako Kozuka, Kumiko Yahikozawa, Koichiro Kuwahara

**Affiliations:** 1 Department of Cardiovascular Medicine, Minaminagano Medical Center, Shinonoi General Hospital, Ai 666-1 Shinonoi, Nagano, 388-8004 Japan; 2 Department of Cardiovascular Medicine, Shinshu University School of Medicine, Matsumoto, Japan

**Keywords:** HFpEF, DBP, Heart failure readmission, Diastolic blood pressure

## Abstract

Although systolic blood pressure (SBP) is routinely considered when treating acute heart failure (HF), diastolic blood pressure (DBP) is hardly been assessed in the situation. There are no previous studies regarding the predictive value of DBP in elderly patients with HF with preserved ejection fraction (HFpEF) in Japan. This study aimed to investigate the prognostic significance of DBP in patients with acute decompensated HFpEF. We analyzed data of all HFpEF patients admitted to Shinonoi General Hospital for HF treatment between July 2016 and December 2018. We excluded patients with acute coronary syndrome and severe valvular disease. Patients were divided into two groups according to their median DBP; the low DBP group (DBP ≤ 77 mmHg, *n* = 106) and the high DBP group (DBP > 77 mmHg, *n* = 100). The primary outcome was HF readmission. In 206 enrolled patients (median 86 years), during a median follow-up of 302 days, the primary outcome occurred in 48 patients. The incidence of HF readmission was significantly higher in the low DBP group (33.0% vs 18.5%, *p* = 0.024). In Kaplan–Meier analysis, low DBP predicted HF readmission (Log-rank test, *p* = 0.013). In Cox proportional hazard analysis, low DBP was an independent predictor of HF readmission after adjustment for age, sex, SBP, hemoglobin, serum albumin, serum creatinine, B-type natriuretic peptide, renin-angiotensin system inhibitors, calcium channel blockers, left ventricular ejection fraction, coronary artery disease, and whether they live alone (hazard ratio, 2.229; 95% confidence interval, 1.021–4.867; *p* = 0.044). Low DBP predicted HF readmission in patients with HFpEF.

## Introduction

Improvements in cardiovascular survival rates and progressive aging of the population have led to an increase in elderly patients with heart failure with preserved ejection fraction (HFpEF) [1–4]. The number of elderly HFpEF patients will continue to increase annually in Japan. Given the high cost of inpatient heart failure (HF) treatment, HF places a major burden on the public health system and has an associated economic impact. Furthermore, repeating HF readmission could worsen the activity of daily living (ADL) and quality of life (QOL) in elderly HF patients. Considering these facts, preventing HF readmission in elderly HF patients would be helpful for both patients and the economic cost.

The fundamental pathophysiological mechanism of HFpEF remains undefined, and the tools for risk-stratification are needed to improve the management of these patients. In clinical practice, we encounter elderly patients with low diastolic blood pressure (DBP) in HFpEF. Decreased DBP is demonstrated to have an association with arterial stiffening, as implied by Sleight’s hypothesis many years ago [5]. The relation between DBP and cardiovascular events could be attributed to the decline in DBP as a consequence of stiffing of the large arteries in elderly patients with HFpEF. Although systolic blood pressure (SBP) is routinely considered when treating acute heart failure [6], DBP is hardly been assessed in the situation. Several recent studies have reported the association between low DBP and adverse outcomes in stable HFpEF without adjusting for SBP [7, 8]. However, there are no previous studies regarding the predictive value of low DBP on HF readmission in an elderly Japanese HFpEF cohort. Against this background, we aimed to identify the prognostic significance of low DBP in elderly patients with acute decompensated HFpEF in a retrospective cohort study.

## Materials and methods

### Study design

**Fig. 1:**
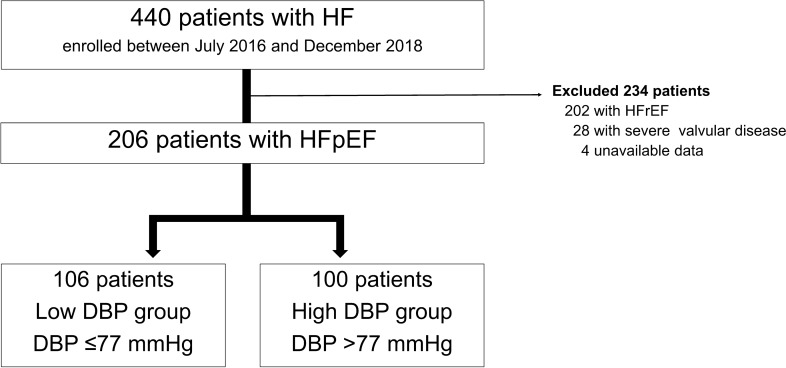
Patient flow chart

This was a retrospective, single-center cohort study. The cohort included patients admitted to Shinonoi General Hospital between July 2016 and December 2018 with a primary diagnosis of acute decompensated HFpEF. Patients with acute coronary syndrome, HF with reduced ejection fraction, and severe valvular heart disease were excluded (Fig. 1). The study and all its protocols were approved by the Shinonoi General Hospital Ethics Committee, and informed consent was obtained. We collected data on clinical characteristics, medical history, major risk factors for HF, comorbidities, laboratory tests, electrocardiography, echocardiography, available angiographic data, medications, treatment and clinical events during hospitalization, and post-discharge follow-up findings.

Blood pressure was defined as the average of two data in sinus rhythm, five data in atrial fibrillation measured in oscillometric method collected at admission, either in the upright or supine position. Acute decompensated HF was defined by the Framingham criteria [9]. The diagnosis of HF and acute coronary syndrome was made by the treating clinicians using all available symptoms, laboratory, electrocardiogram, echocardiography, and coronary angiogram data. Transthoracic echocardiography was performed using standardized equipment (HD15 Ultrasound Machine, Royal Philips, Amsterdam, Noord-Holland, the Netherlands; CX50 xMatrix, Royal Philips, Amsterdam, Noord-Holland, the Netherlands; Toshiba Artida, CANON Medical Systems Corporation, Otawara, Tochigi, Japan) in compliance with the recommendations of the American Society of Echocardiography and the European Association of Cardiovascular Imaging [10, 11]. The biplane modified Simpson’s method was used to measure left ventricular ejection fraction (LVEF). Echocardiography was produced within 24 h after admission, and HFpEF was defined as HF with LVEF ≥ 50%.

All data were fully anonymized before access. The investigation is consistent with the principles outlined in the Declaration of Helsinki.

### Follow-up

The primary outcome was hospitalization during follow-up due to worsening HF. Incidents were validated by chart view.

### Statistical analysis

Continuous variables are summarized as the mean ± standard deviation if normally distributed and as the median and interquartile range if non-normally distributed. Normality was assessed using the Shapiro–Wilk *W*-test. Comparisons of baseline characteristics were made with a contingency table and the Pearson *χ*^2^ test for categorical variables, the *t* test for normally distributed continuous variables, and Mann Whitney *U* test for non-normally distributed continuous variables. Spearman’s rank correlation method was used as a nonparametric measure of the association between DBP and clinical indices. Patients were then divided into two groups according to the median DBP level: the high DBP group (DBP > 77 mmHg, *n* = 100), and the low DBP group (DBP ≤ 77 mmHg, *n* = 106). Kaplan–Meier survival plots were calculated from baseline to the time of HF readmission and compared using the log-rank test. Cox proportional-hazards analysis was used to evaluate the independent prognostic utility of DBP. The covariates used were age, sex, SBP, hemoglobin, serum albumin, serum creatinine, B-type natriuretic peptide (BNP), renin-angiotensin system inhibitors (RASi), calcium channel blockers (CCB), LVEF, coronary artery disease (CAD), and whether they live alone. A *p*-value of < 0.05 was considered statistically significant. All statistical analyses were performed using SPSS Statistics software for Windows Version 26 (IBM Corp., Armonk, NY, USA).

## Results

### Study population

We enrolled 206 patients (median age 86 years; female, 58%). Twenty patients died during hospitalization. The baseline characteristics are shown in Table 1. Patients with low DBP had lower SBP, heart rate, body mass index, BNP, hemoglobin, and higher serum creatinine than those with high DBP. Anticoagulants were more frequently prescribed in the low DBP group. After analysis, there were no strong correlations between DBP and clinical indices (Table 2).

**Table 1:** Baseline characteristics

Variables	Overall population	DBP ≤ 77 mmHg	DBP > 77 mmHg	*p*-value
	(*n* = 206)	(*n* = 106)	(*n* = 100)	
Age (years) [range]	86 [81–91]	86 [81–91]	87 [81–92]	0.976
Male, sex, *n* (%)	87 (42)	48 (45)	39 (39)	0.362
BMI	22.5 [19.6–25.5]	22.0 [19.3–24.6]	23.5 [20.8–25.9]	0.007
Systolic blood pressure (mmHg)	137 [115–154]	120 [102–142]	146 [134–166]	< 0.001
Pulse pressure (mmHg)	55 [42–69]	58 [43–71]	54 [40–63]	0.243
Heart rate (bpm)	85 [70–104]	78 [65–95]	90 [72–110]	0.001
CAD, *n* (%)	43 (21)	22 (21)	21 (21)	0.965
Hypertension, *n* (%)	134 (65)	65 (61)	69 (69)	0.248
Atrial fibrillation, *n* (%)	130 (63)	73 (69)	57 (57)	0.078
Dyslipidemia, *n* (%)	64 (31)	29 (27)	35 (35)	0.236
Diabetes mellitus, *n* (%)	52 (25)	23 (22)	29 (29)	0.228
CKD, *n* (%)	54 (26)	33 (31)	21 (21)	0.098
Living alone, *n* (%)	28 (14)	14 (13)	14 (14)	0.868
Dementia, *n* (%)	62 (30)	30 (28)	32 (32)	0.563
Echocardiographic data				
LVEF (%)	64 [60–68]	64 [60–68]	64 [60–67]	0.653
E/A	1.027 [0.715–1.687]	0.862 [0.696–1.718]	1.072 [0.813–1.670]	0.517
Mean E/e'	16.26 [11.35–21.79]	15.43 [9.426–21.58]	16.70 [13.24–22.67]	0.925
Laboratory data				
BNP (pg/mL)	493 [310–831]	453 [277–673]	507 [360–934]	0.151
Hemoglobin (g/dL)	11.1 [9.7–12.4]	10.8 [9.2–12.1]	11.5 [9.9–12.6]	0.006
Serum albumin (g/dL)	3.4 [3.1–3.8]	3.4 [3.0–3.7]	3.5 [3.1–3.8]	0.106
Serum creatinine (mg/dL)	1.01 [0.73–1.44]	1.11 [0.78–1.62]	0.91 [0.68–1.27]	0.018
HbA1c (%)	5.9 [5.6–6.4]	5.9 [5.5–6.4]	5.9 [5.6–6.4]	0.594
CRP (mg/dL)	0.55 [0.15–2.23]	0.64 [0.17–2.63]	0.47 [0.13–1.52]	0.087
Medication				
Antiplatelet drug, *n* (%)	57 (31)	27 (29)	30 (33)	0.566
Anticoagulant, *n* (%)	101 (54)	58 (62)	43 (47)	0.041
RASis, *n* (%)	113 (61)	55 (59)	58 (63)	0.527
Beta-blockers, *n* (%)	112 (60)	54 (57)	58 (63)	0.436
MRAs, *n* (%)	98 (53)	45 (48)	53 (58)	0.184
Loop diuretic, *n* (%)	156 (84)	77 (82)	79 (86)	0.463
CCBs, *n* (%)	81 (44)	44 (47)	37 (40)	0.365
Statin, *n* (%)	46 (25)	22 (23)	24 (26)	0.672

Values are presented as the mean ± SD, median [interquartile range], or n (%)

*DBP* diastolic blood pressure, *BMI* body mass index, *CAD* coronary artery disease, *CKD* chronic kidney disease, *LVEF* left ventricular ejection fraction, *E/A* early diastolic filling velocity/atrial filling velocity ratio, *E/e'* early diastolic filling velocity/early diastolic velocity of the mitral annulus ratio, *BNP* B-type natriuretic peptide, *CRP* C-reactive protein, *RASi* renin-angiotensin system inhibitor, *MRA* mineralocorticoid receptor antagonist, *CCB* calcium channel blocker

**Table 2:** Univariate Spearman’s rank correlation between diastolic blood pressure and clinical indices

Variables	Spearman’s Rank	*p*-value
Age (years)	0.029	0.680
Male, sex	− 0.064	0.364
Systolic blood pressure (mmHg)	0.480	< 0.001
Hemoglobin (g/dL)	0.183	0.009
Serum albumin (g/dL)	0.120	0.089
Serum creatinine (mg/dL)	− 0.165	0.017
BNP (pg/mL)	0.155	0.028
RASis, *n* (%)	0.046	0.529
CCBs, *n* (%)	− 0.066	0.367
LVEF (%)	− 0.018	0.793
CAD, *n* (%)	0.003	0.966
Living alone, *n* (%)	0.012	0.869

*BNP* B-type natriuretic peptide, *RASi* renin-angiotensin system inhibitor, *CCB* calcium channel blocker, *LVEF* left ventricular ejection fraction, *CAD* coronary artery disease

### The prognostic significance of diastolic blood pressure

**Fig. 2:**
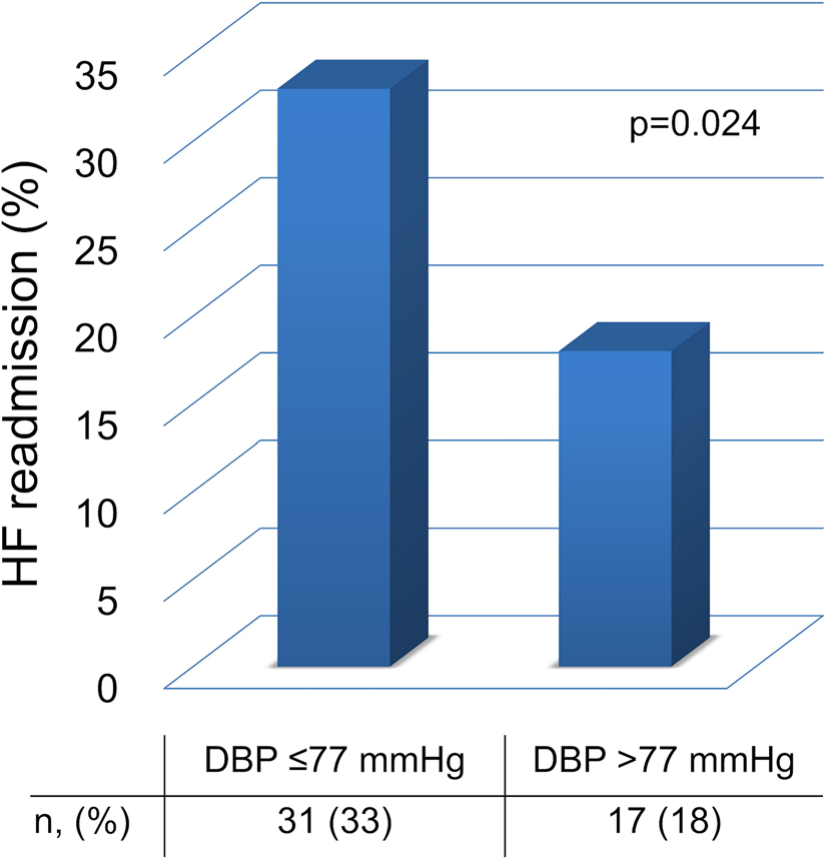
Incidence of heart failure readmission according to diastolic blood pressure level—low diastolic blood pressure was related to an increased risk of heart failure readmission in patients with heart failure with preserved ejection fraction

**Fig. 3:**
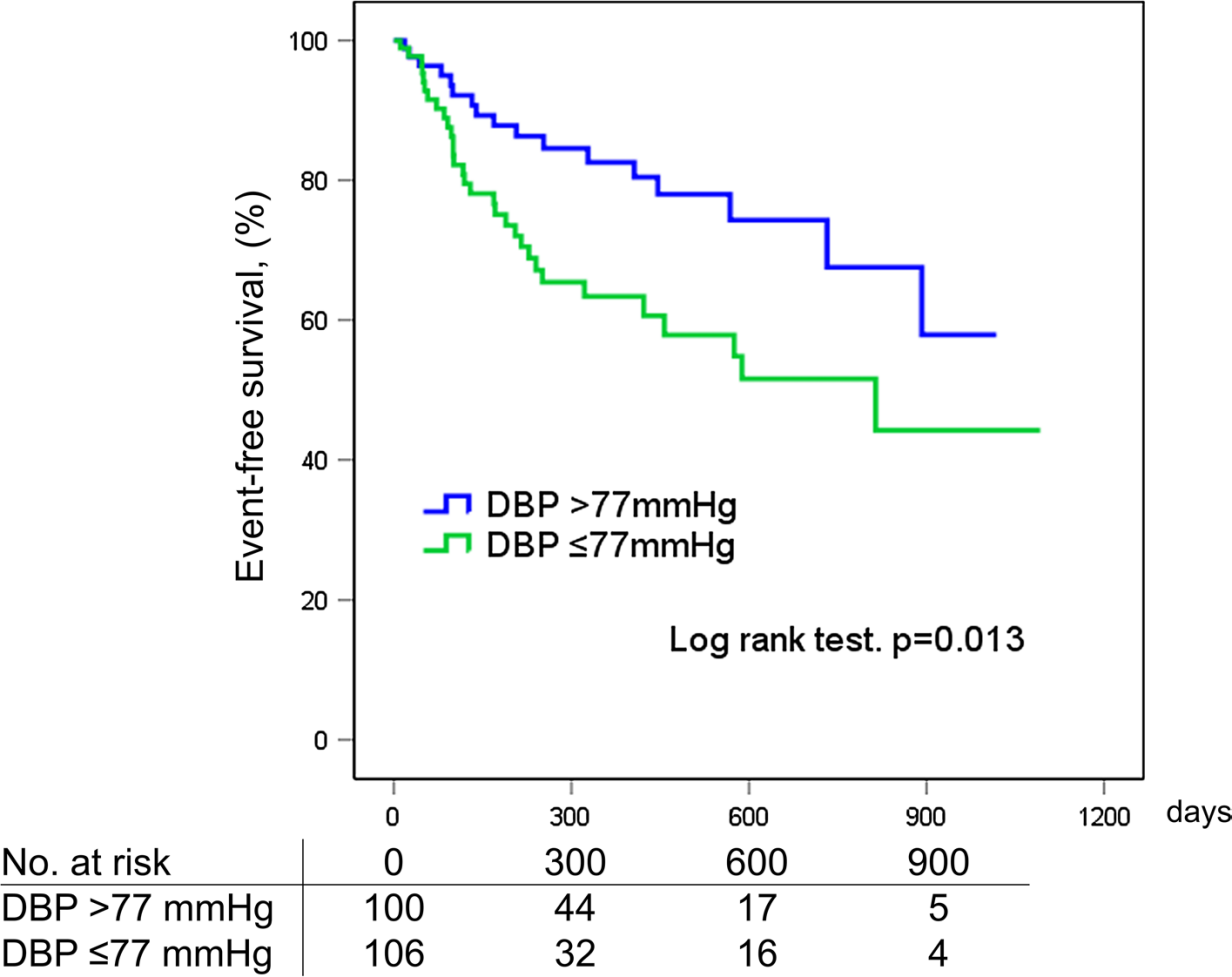
Kaplan–Meier analysis of diastolic blood pressure in patients with heart failure with preserved ejection fraction—low diastolic blood pressure (DBP ≤ 77 mmHg) predicted HF readmission (green line). Blue line, DBP > 77 mmHg

During a median follow-up of 302 days [interquartile range 119–636], 48/186 (25.8%) patients experienced HF readmission. The low DBP group was related to an increased risk of HF readmission [low DBP group 33.0% (31/94) vs high DBP group: 18.5% (17/92), *p* = 0.024] (Fig. 2). In the Kaplan–Meier analysis, the low DBP group predicted HF readmission (Log rank test, *p* = 0.013) (Fig. 3). In the multivariate Cox proportional hazards analysis, the low DBP group was an independent predictor of HF readmission after adjustment for age, sex, SBP, hemoglobin, serum albumin, serum creatinine, BNP, RASi, CCB, LVEF, CAD, and whether they live alone (hazard ratio 2.229; 95% confidence interval 1.021–4.867; *p* = 0.044) (Table 3). The result was consistent upon the exclusion of CAD (hazard ratio 2.229; 95% confidence interval 1.021–4.866; *p* = 0.044).

**Table 3:** Multivariable Cox proportional hazard analysis

Variables	Hazard ratio (95% CI)	*p*-value
Diastolic blood pressure ≤ 77 mmHg	2.229 (1.021–4.867)	0.044
Age (years)	1.019 (0.973–1.068)	0.420
Male, sex	1.459 (0.702–3.039)	0.310
Systolic blood pressure (mmHg)	0.987 (0.973–1.001)	0.072
Hemoglobin (g/dL)	1.021 (0.889–1.173)	0.765
Serum albumin (g/dL)	0.807 (0.400–1.627)	0.548
Serum creatinine (mg/dL)	1.092 (0.629–1.898)	0.754
BNP (pg/mL)	1.000 (0.999–1.001)	0.527
RASis, *n* (%)	0.996 (0.503–1.969)	0.990
CCBs, *n* (%)	0.744 (0.360–1.538)	0.425
LVEF (%)	0.981 (0.928–1.036)	0.490
CAD, *n* (%)	1.006 (0.465–2.178)	0.988
Living alone, *n* (%)	1.213 (0.359–1.865)	0.646

*BNP* B-type natriuretic peptide, *RASi* renin-angiotensin system inhibitor, *CCB* calcium channel blocker, *LVEF* left ventricular ejection fraction, *CAD* coronary artery disease, *CI* confidence interval

## Discussion

The novel finding of the present study is that the low DBP group was significantly associated with an increased risk of HF readmission in extremely elderly patients with acute decompensated HFpEF. This association was independent of other well-established HF risk factors, including age, BNP, renal function, serum albumin, LVEF, and importantly, SBP.

Previous studies have reported the association between low SBP and adverse outcomes in patients with HF [12, 13]. In terms of DBP, several recent studies have investigated the significant association between low DBP and poor prognosis in stable HFpEF [7, 8]. However, these studies were evaluated without adjusting for SBP, and the independent prognostic value of DBP remained unclear.

In our study, we identified that the low DBP group had a significantly higher risk of HF readmission compared with the high DBP group in elderly HFpEF patients hospitalized for acute decompensated HF. To the best of our knowledge, no other study has investigated the prognostic impact of low DBP independent of SBP in these patients.

The underlying pathophysiology of HFpEF remains unclear. A previous study reported that atrial stiffness, a result of the substantial progression of atherosclerosis, could be one of the complex mechanisms of this disease [14–16]. On the other hand, decreased DBP has been demonstrated to indicate arterial stiffening, associated with atherosclerotic progression [5, 17–19]. There is a possibility that large artery stiffening, a result of the substantial progression of atherosclerosis, could be the underlying pathophysiological mechanism of poor prognosis in elderly HFpEF patients with decreased DBP. In addition, low DBP could lead to decreased coronary perfusion pressure, which may result in myocardial damage and worsening ventricular dysfunction [8, 20, 21]. This may also be a reason for the poor prognosis in HFpEF patients with low DBP. However, these hypotheses are only speculative, and further studies are needed.

From our findings, we suggest that DBP could be a useful risk-stratification tool in cases of HFpEF. Although SBP is routinely considered when treating acute heart failure, as cardiac shock or low output syndrome defined by low SBP, DBP is hardly been assessed in the situation. We hypothesize that patients with decreased DBP could have a poor prognosis even if their SBP is preserved. Patients with low DBP at admission should receive aggressive therapy and close outpatient follow-up after discharge. However, although decreased DBP may result in an increased risk of HF readmission, this does not necessarily mean that extremely high DBP are preferred in these patients, since several previous studies have reported that high DBP was, likewise to low DBP, associated with adverse outcomes in patients with cardiovascular disease [8, 22, 23].

Our study had several limitations. First, we included a small number of patients taken from a single center, and the follow-up period was short. Further research in a large cohort is necessary to verify our findings. Second, there was only a single data of DBP at the decompensated phase of HF. Serial changes or continuous measures of DBP were not evaluated in this study, and DBP measures could have changed after measurement. Third, we diagnosed HFpEF according to the echocardiographic data obtained within 24 h after admission. We did not have data at the compensated phase of HF, which may have affected the patient cohort.

## Conclusion

Low DBP was independently associated with HF readmission in hospitalized patients with decompensated HFpEF. Our findings suggest that DBP may be a useful risk-stratification tool in this population.
